# Association between polymorphism rs2421943 of the insulin‐degrading enzyme and schizophrenia: Preliminary report

**DOI:** 10.1002/jcla.24949

**Published:** 2023-07-28

**Authors:** Laura Ambrozová, Tomáš Zeman, Vladimír Janout, Jana Janoutová, Jan Lochman, Omar Šerý

**Affiliations:** ^1^ Laboratory of Neurobiology and Molecular Psychiatry Department of Biochemistry Faculty of Science Masaryk University Brno Czech Republic; ^2^ Laboratory of Neurobiology and Pathological Physiology Institute of Animal Physiology and Genetics Czech Academy of Sciences Brno Czech Republic; ^3^ Department of Public Health Faculty of Medicine and Dentistry Palacky University Olomouc Czech Republic

**Keywords:** candidate gene analyses, genetic association study, insulin‐degrading enzyme (IDE), miRNA, schizophrenic disorder, single nucleotide polymorphism (SNP)

## Abstract

**Background:**

Insulin‐degrading enzyme (IDE) is an important gene in studies of the pathophysiology of type 2 diabetes mellitus (T2DM). Recent studies have suggested a possible link between type 2 diabetes mellitus (T2DM) and the pathophysiology of schizophrenia (SZ). At the same time, significant changes in insulin‐degrading enzyme (*IDE*) gene expression have been found in the brains of people with schizophrenia. These findings highlight the need to further investigate the role of *IDE* in schizophrenia pathogenesis.

**Methods:**

We enrolled 733 participants from the Czech Republic, including 383 patients with schizophrenia and 350 healthy controls. Our study focused on the single nucleotide polymorphism (SNP) rs2421943 in the *IDE* gene, which has previously been associated with the pathogenesis of Alzheimer's disease. The SNP was analyzed using the PCR‐RFLP method.

**Results:**

The G allele of the rs2421943 polymorphism was found to significantly increase the risk of developing SZ (*p* < 0.01) when a gender‐based analysis showed that both AG and GG genotypes were associated with a more than 1.55 times increased risk of SZ in females (*p* < 0.03) but not in males. Besides, we identified a potential binding site at the G allele locus for has‐miR‐7110‐5p, providing a potential mechanism for the observed association.

**Conclusion:**

Our results confirm the role of the *IDE* gene in schizophrenia pathogenesis and suggest that future research should investigate the relationship between miRNA and estrogen influence on *IDE* expression in schizophrenia pathogenesis.

## Graphical abstract

We genotyped IDE polymorphism rs2421943 in 383 patients with schizophrenia and 350 healthy controls. We identified a significant increase in schizophrenia risk associated with the G allele and GG genotype in females and identified a potential binding site at the G allele locus for miRNA has‐miR‐7110‐5p providing a potential mechanism for the observed association.

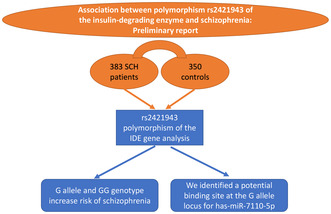

## 1 INTRODUCTION

Schizophrenia (SZ) is a serious neuropsychiatric disorder with a global prevalence of approximately 0.5%–1%, often associated with persistent symptoms, the need for antipsychotic medication, and hospitalization.^1^, ^2^ This neurodevelopmental disorder is characterized by profound disturbances in cognition and perception, inappropriate emotionality, and impaired ability to behave appropriately.^3^ The higher prevalence of cardiovascular disease, the leading cause of early mortality in patients with schizophrenia, is often attributed to the adverse effects of antipsychotic medications^4^ or general lifestyle.^5^ Based on this fact, some authors have suggested that type 2 diabetes mellitus (T2DM) in patients with SZ may be a side effect of this antipsychotic medication, especially of the atypical antipsychotics, which cause rapid weight gain.^6^ However, recent meta‐analyses of case–control studies suggest that insulin resistance, which is a significant risk factor for T2DM and obesity,^7^, ^8^ is also associated with relatively young antipsychotic‐naïve patients with first‐episode psychosis,^9^, ^10^ and thus may be a causally related to or share pathophysiological mechanisms with schizophrenia. Furthermore, molecular inference and GWAS studies also suggest that SZ shares a substantial polygenic component with T2DM.^11^ Liu et al.^12^ provide a general pathway‐based view of the pathogenetic association between these two diseases and identified a set of 33 highly significant genes associated with both SZ and T2DM. These genes include genes involved in both cognitive function and glucose metabolism that previous studies have suggested may increase the risk of T2DM in schizophrenia patients and vice versa, including the AKT signaling pathway, which plays a key role in insulin metabolism in the liver.^12^

Insulin‐degrading enzyme (IDE) plays a crucial role in studies of T2DM. The *IDE* gene is located on chromosome 10q23.33 in humans and encodes a zinc‐binding, neutral, thiol metalloprotease, whose association with proteasome components has recently been demonstrated.^13^ IDE also prevents peptide aggregation by cleaving several peptides, including insulin, β‐amyloid protein, and glucagon,^14^, ^15^ as well as other diverse peptides, such as insulin‐like growth factors I and II (IGF‐I and II)^16^ or β‐endomorphin.^17^ *IDE* is expressed in various organs and tissues, with its expression being highest during development and decreasing with age, particularly in the brain.^18^ This age‐associated impairment of IDE may explain the accumulation of some of these amyloidogenic proteins, either because they are direct proteolytic substrates of IDE.^19^ Besides, IDE is specific for β‐sheet substrates,^20^ making its proteolytic activity relevant against toxic oligomer formation associated with neurodegenerative diseases such as Alzheimer's disease (AD) and Parkinson's disease (PD). Given that insulin can modulate the clearance of extracellular Ab oligomers by regulating IDE, it represents a key molecular link between AD, PD, and T2DM today.^21^, ^22^, ^23^ However, some recent studies suggest the possible involvement of the IGF‐I pathway in SZ, along with disturbed insulin signaling pathways, and suggest an association of T2DM with SZ in drug‐naïve patients, providing a reason to believe that brain IDE may be involved in SZ.^18^, ^24^ The expression of IDE has been found to be significantly reduced in the dorsolateral prefrontal cortex of patients with schizophrenia compared with controls, suggesting a possible role in the disturbed insulin signaling cascades and altered neuropeptide metabolism in the disease.^18^

Previous studies have shown an association between the *IDE* gene polymorphisms rs1887922 and rs2149632 and T2DM.^25^, ^26^ However, to date, no study has investigated whether *IDE* gene SNPs are associated with an increased risk of SZ. Therefore, this study aimed to investigate the association of polymorphism (SNP) rs2421943 of the *IDE* gene, previously associated with an increased risk of AD and mild cognitive impairment (MCI),^22^ with SZ using a case–control association approach.

## 2 MATERIALS AND METHODS

### 2.1 Ethics statement

The study was conducted in accordance with the Helsinki Declaration of 1975 (revised 2000) and approved by the Ethics Committee of the Faculty of Medicine, University of Ostrava (08/2012). Informed consent was obtained from all participants or their legal representatives.

### 2.2 Participants

In our case–control study, we enrolled a total of 733 participants, including 383 patients with schizophrenia (SZ) and 350 healthy controls from the Czech Republic who gave informed consent. Patients with schizophrenia were recruited from the Department of Psychiatry, Faculty Hospital in Brno, and the Psychiatric Hospital in Jihlava. The diagnosis of SUD was made by qualified psychiatrists according to the criteria of the International Classification of Diseases, 11th edition (ICD‐11), and the Diagnostic and Statistical Manual of Mental Disorders, 5th edition (DSM‐V). Patients with psychiatric comorbidity were excluded from the study. The control group consisted of volunteers recruited from various sources, including blood donors from the Brno Blood Bank, patients treated for erectile dysfunction at the Trauma Hospital in Brno, employees of private companies in Brno, farms in the Brno area, university academics, and employees of the National Theatre in Brno. Each member of the control group underwent the Mini‐International Neuropsychiatric Interview (M.I.N.I.,^27^) to screen for psychiatric disorders, and individuals suspected of not being fully mentally healthy were excluded from the group. All screening and interviews were conducted by two qualified psychiatrists with similar backgrounds who communicated closely with each other or, in the case of controls, by one of the authors (O.S.) assisted by a qualified psychologist to minimize personal bias. Blood samples were taken from all participants, and descriptive statistics of the patients and controls included in the study are shown in Table 1.

**TABLE 1:** Descriptive statistics of the patients diagnosed with SZ and respective controls with similar age distribution.

Variable	Female	Female	Male	Male
	Patients	Controls	Patients	Controls
	(*N* = 124)	(*N* = 180)	(*N* = 259)	(*N* = 170)
Age	45.13 ± 14.08	44.58 ± 13.88	37.7 ± 10.86	38.25 ± 12.46
Age of diagnosis	25.23 ± 9.69		24.62 ± 7.25	
Highest education achieved				
Primary education	30 (24%)	14 (8%)	64 (25%)	9 (5%)
Secondary education	88 (71%)	134 (74%)	176 (68%)	104 (61%)
Tertiary education	6 (5%)	32 (18%)	19 (7%)	57 (34%)
Married	15 (12%)	58 (32%)	18 (7%)	83 (49%)
Employed	11 (9%)	104 (58%)	29 (11%)	126 (74%)

*Note*: Mean ± standard deviation is given for age distribution.

### 2.3 Genetic analysis

Genomic DNA was isolated from EDTA containing whole blood samples using the EliGene® Blood DNA Isolation Kit (Elisabeth Pharmacon). The DNA was amplified via polymerase chain reaction (PCR) using the Veriti thermal cycler (Applied Biosystems). The selected SNP rs2421943 (chr10:92552058), which has a minor allele frequency of 0.44 in the European population, was analyzed using the PCR‐RFLP method. The fragment with analyzed SNP was amplified using previously designed primers 5′‐ TGAGTTATTATTTGATGGGTACAGA‐3′ as a forward (left) primer and 5′‐GTTTGTTAGAATATTGATTGTTTCTGA‐3′ as a reverse (right) primer.^22^ The PCR reaction mixture contained 1 μL of DNA template, primers at a concentration of 0.25 μM, and EliZyme HS FAST MIX 2x (Elisabeth Pharmacon, Czech Republic) in a final volume of 20 μL. The initial denaturation was carried out for 3 min at 95°C, followed by 35 cycles of 95°C for 10 s, 56°C for 30 s, and 72°C for 20 s, with a final extension at 72°C for 5 min. The resulting PCR amplicon of 370 bp was digested with *Mbo*I restriction endonuclease (ER0812, Thermo Fisher Scientific) for 16 h at 37°C and then separated on a 2.5% agarose gel stained with ethidium bromide. The presence of the A allele yielded 3 fragments with lengths of 29, 145, and 196 bp, while the G allele produced two fragments with lengths of 174 and 196 bp.

### 2.4 Statistical and in silico analysis

Statistical analysis was performed using R software.^28^ Fisher's exact test was used to assess the association between discrete variables, including disease onset, genotype, cannabis use, and drug use. The risk ratio (RR) and odds ratio (OR) were used to determine the differences between genotypes or alleles in the calculated risk and odds. The confidence interval (CI) for each OR was calculated using the logit method as previously described.^29^ The R package *statmod* ^30^ was used to calculate the power of Fisher's exact test by simulation.

We also performed an analysis of the potential involvement of the intronic rs2421943 polymorphism in the enhancer regulatory element of neighboring genes using the GeneHancer database and in miRNA regulation using miRBase and mirDIP.

## 3 RESULTS

Table 1 presents the descriptive statistics for both patients and controls. We found that in the control group, the A allele had a frequency of 0.48 and the G allele a frequency of 0.52, with genotype frequencies of 0.25 for AA, 0.45 for AG, and 0.30 for GG. Among patients diagnosed with schizophrenia, the A allele had a frequency of 0.41 and the G allele a frequency of 0.59, with genotype frequencies of 0.19 for AA, 0.45 for AG, and 0.36 for GG. The rs2421943 polymorphism of the *IDE* gene was found to be associated with SZ risk using Fisher's exact test. The G allele was found to significantly increase the risk of developing SZ (*p* < 0.01) compared with the A allele in subjects. The GG genotype was also found to be associated with a significant increase in SZ risk (*p* = 0.0141) with a risk of over 1.29 times that of the A allele. Notably, the statistically significant risk was observed only in homozygotes (GG – *p* < 0.0141), while heterozygotes (AG) did not exhibit a significant risk of SZ (Table 2).

**TABLE 2:** Association between rs2421943 polymorphism and schizophrenia.

Genotype/Allele	*N*		Risk	RR	Odd	OR (95% CI)	*p*
	SZ	Controls					
AA	71	88	0.45		0.81		
AG	172	159	0.52	1.16	1.08	1.33 (0.91–1.94)	0.1478
GG	140	103	0.58	1.29	1.36	1.68 (1.12–2.51)	**0.0141**
A	314	335	0.48		0.94		
G	452	365	0.55	1.15	1.24	1.32 (1.11–1.53)	**0.0085**
AA or AG	243	247	0.5		0.98		
GG	140	103	0.58	1.16	1.36	1.39 (1.02–1.89)	**0.0416**
AA	71	88	0.45		0.81		
AG or GG	312	262	0.54	1.2	1.19	1.47 (1.03–2.09)	**0.0316**

*Note: p* values in bold signify a difference statistically significant at *p* < 0.05.

Abbreviations: CI, confidence interval; *N*, number of subjects; OR, odds ratio; SZ, schizophrenia.

Moreover, a gender‐based analysis showed that both AG and GG genotypes of the rs2421943 polymorphism were associated with a 1.55 times increased risk of SZ in females, indicating a dominant effect of the G allele (*p* < 0.03). The power of the tests, calculated based on the observed risks and sample sizes, was higher than 0.56 in all comparisons that found a statistically significant difference in schizophrenia risk between genotypes. No statistically significant association was observed between SZ and any genotype in males (Table 3). Comparisons of observed and expected genotype frequencies in our study group under Hardy–Weinberg equilibrium are shown in Table 4. Genotype frequencies in the control and patients group were consistent with the Hardy–Weinberg equilibrium (*p* > 0.05).

**TABLE 3:** Association between rs2421943 polymorphism and schizophrenia according to sex. P values in bold signify a difference statistically significant at P<0.05.

Sex	Genotype/Allele	*N*		Risk	RR	Odd	OR (95% CI)	*p*
		SZ	Controls					
Female	AA	22	55	0.29		0.4		
	AG	57	70	0.45	1.55	0.81	2.02 (1.1–3.7)	**0.0260**
	GG	45	55	0.45	1.55	0.82	2.05 (1.09–3.86)	**0.0292**
	A	101	180	0.36		0.56		
	G	147	180	0.45	1.25	0.82	1.46 (1.14–1.79)	**0.0256**
	AA or AG	79	125	0.39		0.63		
	GG	45	55	0.45	1.15	0.82	1.3 (0.8–2.11)	0.3215
	AA	22	55	0.29		0.4		
	AG or GG	102	125	0.45	1.55	0.82	2.05 (1.17–3.59)	**0.0154**
Male	AA	49	33	0.6		1.48		
	AG	115	89	0.56	0.93	1.29	0.87 (0.52–1.46)	0.6919
	GG	95	48	0.66	1.1	1.98	1.34 (0.76–2.35)	0.3174
	A	213	155	0.58		1.37		
	G	305	185	0.62	1.07	1.65	1.2 (0.93–1.48)	0.2048
	AA or AG	164	122	0.57		1.34		
	GG	95	48	0.66	1.16	1.98	1.48 (0.97–2.25)	0.0756
	AA	49	33	0.6		1.48		
	AG or GG	210	137	0.61	1.02	1.53	1.03 (0.63–1.69)	0.9007

*Note: p* values in bold signify a difference statistically significant at *p* < 0.05.

Abbreviations: CI, confidence interval; *N*, number of subjects; OR, odds ratio.

**TABLE 4:** Comparisons of observed and expected genotype frequencies under Hardy–Weinberg equilibrium (HWE).

Genotype	Observed frequencies	Expected frequencies under HWE
AA	0.2169	0.1960
AG	0.4516	0.4934
GG	0.3315	0.3106

To investigate the possible role of intronic rs2421943 polymorphism, we performed an analysis of its potential involvement in the enhancer regulatory element of nearby genes using the GeneHancer database,^31^ as well as an analysis of miRNA regulation with miRBase for mature miRNAs specific to humans^32^ and mirDIP.^33^ Our findings indicated no presence of enhancer regulatory elements at the polymorphism site. However, we did find that the G allele was the target of the has‐miR‐7110‐5p miRNA, as identified by both miRbase and miDIP. To further support this observation, we employed the RNA22 v2 program^34^ to analyze parameters of the hsa‐miR‐7110‐5p interaction, using a sensitivity of 45% and specificity of 78%, with a seed size of 7 and a maximum of 1 unpaired base in the seed. The analysis indicated a high probability of an interaction site for the hsa‐miR‐7110‐5p molecule in the case of the G allele (Table 5).

**TABLE 5:** Analysis of parameters of the hsa‐miR‐7110‐5p interaction with sequence surrounding the polymorphism rs2421943 by RNA22 v2 software.^34^

	Allele	Folding energy (kcal/mol)	Heteroduplex	*p* Value
hsa_miR_7110_5p	rs2421943 (G)	−32.30	TTTCTCA**C**CCCACCCCCCCA |:|||| |||||| |||||| AGAGAGAGGGGTGTGGGGGT	6.83E‐2
hsa_miR_7110_5p	rs2421943 (A)	−29.90	TTTCTCA**T**CCCACCCCCCCA |:|||| ||||| |||||| AGAGAGAGGGGTGTGGGGGT	4.64E‐2

*Note*: The sequence of the (−) strand of *IDE* is underlined. The alternate bases in the polymorphism are magnified in bold.

## 4 DISCUSSION

In our study, we investigated the association between the rs2421943 polymorphism of the *IDE* gene and the risk of SZ. Although the G allele and GG genotype appear to be risk factors for the predisposition to schizophrenia in the entire study group including both sexes, after dividing the sample into men and women, the G allele and GG genotype remain risk factors only in the subgroup of women. The frequencies of the G allele and GG genotype were 0.59 and 0.36, respectively, among female patients diagnosed with schizophrenia, while in the control group, these frequencies were 0.50 and 0.31, respectively. Interestingly, we observed a high probability of interaction with the miRNA molecule hsa‐miR‐7110‐5p specifically in the presence of the G allele. These results suggest that the *IDE* gene may be partly responsible for the increased risk of SZ in females.

Our results show an association between the *IDE* rs2421943 polymorphism and the risk of SZ only in female patients. Interestingly, a previous study found the same sex‐specific association with postprandial insulin clearance in a European population‐based cohort.^35^ Our findings add to the growing list of sex differences in schizophrenia. These include the significantly later age of onset of SZ in female patients, which has been attributed to the neuroprotective and antidopaminergic properties of estradiol,^36^, ^37^ and the transforming growth factor beta (TGF‐β) pathway involving the TGFB1 + 869 T/C polymorphism in female schizophrenia patients.^38^ Recent studies showed significant sex differences in DNA methylation.^39^ A previous study by Zhao et al.^40^ showed a difference in *IDE* expression in response to estradiol, a female sex hormone. In vitro experiments have shown that 17β‐estradiol increases *IDE* expression at both mRNA and protein levels in a time‐dependent manner, and that the effect is dependent on the estrogen receptor β and requires activation of phosphatidylinositol 3‐kinase. In vivo studies in adult female rats^40^ have shown that *IDE* expression is region‐specific, with a significant decrease in *IDE* expression detected in the hippocampus after ovariectomy, which can be prevented by 17β‐estradiol treatment. Thus, the sex‐specific association between the rs2421943 polymorphism of the *IDE* gene and SZ risk found in our study, particularly in women, may be explained by the effect of estrogen on IDE expression. Further studies are needed to determine the exact mechanisms underlying this association.

The *IDE* gene has been implicated in several diseases, including T2DM, AD, and PD. Polymorphisms within the IDE genomic region have been shown to be associated with an increased risk of T2DM due to the involvement of IDE in the control of systemic insulin levels.^14^ Similarly, there is a well‐described association between Alzheimer's disease and IDE based on Aβ clearance, with the G allele of the rs2421943 polymorphism of the *IDE* gene being associated with an increased risk of Alzheimer's disease and mild cognitive impairment.^22^ To date, no studies have specifically investigated the relationship between polymorphisms of the *IDE* gene and schizophrenia. Interestingly, in the central nervous system, insulin is involved in the intersection of metabolic and cognitive disorders,^41^ and there is a suggested association between T2DM and SZ in drug‐naïve patients.^24^ Many metabolic genes that are downregulated in SZ are regulated by insulin,^42^ and IDE is a downstream target of the insulin receptor signaling pathway. In addition, several linkage analyses have suggested an association between SZ and chromosomal loci between 10q23 and 10q25, where the *IDE* gene is located.^43^, ^44^, ^45^ Previous research has reported a significant reduction in the number of IDE‐expressing neurons and IDE protein levels in post‐mortem brains of SZ patients compared with controls.^18^

Our in silico analysis revealed that the *IDE* rs2421943 polymorphism is located in a region highly likely to be targeted by the miRNA hsa‐miR‐7110‐5p, with the G allele at position 13 increasing the affinity of hsa‐miR‐7110‐5p for this binding site (Table 5). This finding provides a potential explanation for the observed differences in allele and genotype frequencies and their association with SZ risk. The increased affinity of the G allele for hsa‐miR‐7110‐5p may lead to altered gene regulation, potentially contributing to the development of SZ. The G allele of the *IDE* rs2421943 polymorphism may potentially hinder the transition of hnRNA to mRNA, leading to a slower translation of the IDE protein in the brain, which has previously been reported in postmortem brains of SZ patients.^18^ This reduction in IDE enzymatic activity would lead to increased levels of insulin interacting with brain regions involved in cognitive function.^46^, ^47^ In addition, recent in vitro studies have demonstrated the role of miRNAs and RNA‐binding proteins in *IDE* regulation.^48^

This study has some limitations that need to be addressed. First, the sample size was limited. However, we ensured that all patients and controls were accurately diagnosed and attempted to achieve a high level of consistency between the two groups. Second, the results of the miRNA in silico analysis and the resulting interpretations could be further confirmed by examining post‐mortem human brain tissue for potential associations between rs2421943 and IDE (and/or insulin) levels, as well as the presence of hsa‐miR‐7110‐5p. Although the presence of hsa‐miR‐7110‐5p in the cerebrospinal fluid of patients with intracerebral hemorrhage has been demonstrated,^49^ its involvement in the pathogenesis of type 1 diabetes mellitus^50^ suggests that hsa‐miR‐7110‐5p may be present in other parts of the body. In conclusion, our study found a sex‐specific association between the *IDE* rs2421943 polymorphism and SZ risk, with the G allele increasing the risk only in females. Our in silico analysis revealed a potential mechanism by which the G allele may interfere with IDE enzymatic activity through miRNA regulation. However, further studies are needed to confirm these findings and to elucidate the precise mechanisms underlying the association.

## AUTHOR CONTRIBUTIONS

OS, VJ, and JL received a grant project and conceived the design of the study and basic concepts. JL and LA performed laboratory analyses. JJ and OS were responsible for patient recruitment and collection of clinical data. TZ performed the statistical analysis. JL performed in silico analyses. LA, JL, and OS wrote the article. All authors read and approved the final version of the manuscript.

## FUNDING INFORMATION

This work has been supported by the Czech Health Research Council, Czech Republic (AZV ČR) – grant projects No. NT14504 and NV18‐04‐00455, and by the Czech Science Foundation (GAČR) project No. GP309/09/P361.

## CONFLICT OF INTEREST STATEMENT

The authors declare that the research was conducted in the absence of any commercial or financial relationships that could be construed as a potential conflict of interest.

## Data Availability

The raw data supporting the conclusions of this article will be made available by the authors, without undue reservation
